# Our lifestyles are back to ‘normal’, but is our mental health? Longitudinal assessment of psychological distress during the COVID-19 pandemic among Spanish adults: April 2021 to August 2022

**DOI:** 10.1371/journal.pgph.0003389

**Published:** 2024-07-17

**Authors:** Aviana O. Rosen, Maria Dolores Hidalgo, Colleen B. Mistler, Nekane Balluerka, Arantxa Gorostiaga, Juana Gómez-Benito, Ashley L. Holmes, Tania B. Huedo-Medina

**Affiliations:** 1 Department of Allied Health Sciences, University of Connecticut, Storrs, Connecticut, United States of America; 2 Department of Basic Psychology and Methodology, University of Murcia, Murcia, Spain; 3 Department of Clinical and Health Psychology, and Research Methods, School of Psychology, University of the Basque Country UPV/EHU, Donostia, Spain; 4 Group on Measurement Invariance and Analysis of Change (GEIMAC), Institute of Neuroscience, University of Barcelona, Barcelona, Spain; 5 Department of Social Psychology and Quantitative Psychology, Faculty of Psychology, University of Barcelona, Barcelona, Spain; 6 Department of Health Policy, The George Washington University, Washington, D.C, United States of America; London School of Hygiene and Tropical Medicine Faculty of Epidemiology and Population Health, UNITED KINGDOM OF GREAT BRITAIN AND NORTHERN IRELAND

## Abstract

The COVID-19 pandemic had a detrimental effect on mental health since its start in 2020 and current data on mental health is limited. This study provides recent longitudinal data on psychological distress among a country-wide sample of adults. We recruited and surveyed 1,956 adults in Spain in April 2021 and August 2022 on sociodemographic- and pandemic-related psychological distress using the General Distress 21-item version of the Depression, Anxiety, and Stress Scale (DASS). Paired sampled t-tests assessed DASS scores by sex from April 2021 to August 2022; and one-way ANOVAs assessed DASS scores across sociodemographic characteristics. Results showed that psychological distress slightly improved across the total sample from April 2021 to August 2022; though females, young adults, students, and individuals with lower income experienced more psychological distress. Increases in severe stress scores were found particularly among men. Our data provides an overview of the psychological distress of Spanish adults 2.5 years into the pandemic and provides novel evidence that though life has resumed a sense of normalcy after the COVID-19 pandemic, the mental health of key populations (e.g., females, young adults, students, low-income) is still suffering and further intervention and resources are needed.

## Introduction

The World Health Organization (WHO) has recently announced that the COVID-19 pandemic is no longer considered a global health emergency, though the ramifications of the pandemic continue to have a devastating impact on global health and economy [1]. Particularly, the *silent pandemic* is the mental health impact individuals are experiencing worldwide due to socioecological stressors (e.g., lack of social interaction, financial burden, coping with widespread illness and death, changes in routine, among others) that the SARS-CoV-2 virus has exacerbated [2]. In 2022, the WHO announced a 25% global increase in prevalence of depression and anxiety since the start of the pandemic, and worldwide studies corroborate that the increasing prevalence of anxiety, depression, and stress continues to have adverse psychological effects on individuals [2–16]. While many aspects of life have returned to ‘normalcy’, the SARS-CoV-2 virus continues to mutate and rage through the global population, though data from 2022 on the state of mental health more than two years into the pandemic is extremely limited to nonexistent.

Our research team has been collecting data on the psychological distress of adults in Spain since the start of the pandemic in April 2020 [17]. Spain has proven unique in the COVID-19 pandemic, as it was one of the first countries outside of China to see dramatic increases in COVID-19 cases and associated deaths at the start of the pandemic [18]. The Spanish government’s response was unified throughout the country in implementing several strict lockdowns to halt the rapid spread of the virus [18, 19]. These lockdowns were temporarily effective, although traumatic for much of the population [20–23]. As of February 2024, Spain has confirmed about 14 million COVID-19 cases, and approximately 122,000 COVID-related deaths [24]. Spain continued to have several surges in the virus in 2022, though most COVID-related restrictions were lifted.

Data collected from our research team in April 2020 found that a sample of Spanish adults experienced negative changes in attitude and mood during the April 2020 lockdown compared to pre-pandemic [17, 25, 26]. These changes included distress, sadness, depression, anxiety, and worry, among others. Notably, females and young adults (i.e., aged 18–34) reported greater increases in psychological distress, feelings of depression, mood swings, panic attacks, and several other negative psychological symptoms. Our April 2020 data is consistent with other data coming from Spain and globally at the time, although data has been limited since then [6–8, 25, 27–31]. We have since collected data in April 2021 and August 2022 to longitudinally track and assess psychological distress among this sample throughout the pandemic context and outside of the lockdown experience. Data from January 2021 in Spain found that emotional distress had worsened from the beginning of the pandemic [8]; whereas data from January 2022 found that positive mental health and confidence in coping with COVID-19 for females had improved from 2021 but worsened for males [6].

The majority of research from the COVID-19 pandemic, even research published in 2021 through 2023, has data collected during the beginning of the pandemic and first lockdown periods (~April 2020 –summer 2020). Some research with data from 2021 or early 2022 has emerged since, though much of this literature is focused on specific sub-populations (e.g., healthcare workers, college students, individuals with special needs, among others) [3–8, 32–38]. Further, to the best of our knowledge, no current research has been published that is representative of an entire country’s population, particularly longitudinally. Thus, research on the long-term mental health effects of the ongoing pandemic is limited, and as the pandemic situation is constantly transforming, updated data is urgently needed. Therefore, the purpose of this study is to provide recent data from late 2022 on the psychological distress of a representative sample of Spanish adults, approximately two and a half years into the COVID-19 pandemic, and longitudinally assess any changes from 2021. The most recent data from the pandemic suggests that psychological distress (e.g., symptoms of depression, anxiety, stress) continue to worsen or remain at elevated levels, thus we hypothesize that psychological distress will continue to suffer two and a half years into the pandemic [6, 8].

## Materials and methods

### Overview

This paper will provide data from surveys conducted at two critical timepoints of the pandemic in Spain after the lockdown period: April 2021 (Wave 1), one year after the extremely strict mandatory lockdown at the start of the pandemic and vaccine distribution was beginning; and the last week of July into August 2022 (Wave 2), when much of the Spanish population was fully vaccinated (~85.5%) and most pandemic-related restrictions were lifted, though continuous surges in the virus persisted. This study received Institutional Review Board approval from the University of Connecticut (USA), and the University of the Basque Country (Spain).

### Recruitment

We utilized the consumer panelist company Netquest for our recruitment and data collection [39]. Netquest answers and complies with the European Society for Opinion and Market Research’s (ESOMAR) 37 questions and qualifications regarding recruitment, sampling and project management, privacy policies, and metrics [40]. Netquest originated in Spain and now recruits from a global panel of 1.5 million members. As our target sample was adults in Spain, Netquest employed a complex and quality-controlled recruitment procedure to ensure a representative and unique sample in Spain. The key sociodemographic variables of the population were considered as quotas, allowing for representative recruitment by sex, socioeconomic status, autonomous community (i.e., region) of Spain, and age. Only participants under 18 years of age were excluded from participation and thus were not contacted by Netquest for invitation to the study. Netquest sends an email invitation to potential panelists, and participants are compensated by the company with a points-for-reward redemption system. Netquest has several procedures in place to avoid sampling and respondent bias including invitation-only recruitment ensuring engaged and validated participants, a variety of procedures in place to investigate any potential duplicate participants, a router technology system that detects if a participant has already fulfilled a quota for that survey, and only sharing incentive amount after completion [39]. Finally, Netquest ensures high security and data protection by following the national data protection regulations of the countries in which they operate. All participants consent to being a panelist with the company, in addition to consenting to participating in our specific study by electronically signing a consent form before participation in the survey.

Netquest resurveyed a subset of participants from the April 2020 sample: 3,500 for Wave 1 and 1,956 for Wave 2. For Wave 1, all participants who were invited to participate accepted and completed the survey. For Wave 2, 2,500 were invited to participate, and 1,956 accepted and completed the survey. Wave 2 reduced sample size was attributed to several factors: 1) In order to allow for increased participant survey completion time from adding new items to the Wave 2 survey, we had to reduce our sample size to remain within the project budget. 2) Our target sample size for Wave 2 was ~2,000 participants. Netquest detailed attrition estimates and oversampled (2,500) based on the estimates and our target sample size. 3) Due to summer vacations in Spain in August, it was difficult to reach the full recruitment target at the time of Wave 2 recruitment. For this study, we only analyzed the 1,956 participants who completed both the Wave 1 and Wave 2 surveys.

### Data collection and measures

Participants completed a comprehensive survey questionnaire that consisted of COVID-19-related questions assessing areas of lifestyle, behavior, psychological distress, coping strategies, and more. For the purpose of this project, we assessed sociodemographic characteristics such as sex, age, civil status, occupation, and average monthly household income. We measured symptoms of depression, anxiety, and stress using the General Distress 21-item version of the Depression, Anxiety, and Stress Scale (DASS-21) [41]. This 21-item (total) scale measures symptoms of each subscale (i.e., depression, anxiety, stress) through 7 items, consisting of statements such as, in the last 7 days, ‘I have not been able to feel any positive emotion’ (depression), ‘I have felt on the edge of panic’ (anxiety), ‘It has been difficult to relax’ (stress). Responses were collected on a scale of 0 (does not apply to me) to 3 (very applicable to me, or applicable the majority of time). Scores for each subscale are summed, and because the DASS-21 is the shorter version of the original 42-item scale, the score for each subscale is multiplied by two to obtain a total score. A higher score for each subscale represents poorer depression, anxiety, and stress; likewise, a higher total score represents poorer depression, anxiety, and stress combined. Further, subscale scores can be divided into levels of severity ratings comprising of normal, mild, moderate, severe, and extremely severe depression, anxiety, and stress. This scale has been validated in Spanish, among Spanish samples, and for use during the pandemic [42–44]. Reliability for this sample: total DASS Cronbach’s alpha α = .96; depression subscale α = .93; anxiety subscale α = .88; stress subscale α = .90.

### Ethics statement

This research was approved by the Institutional Review Board of the University of Connecticut. Written informed consent was obtained by all study participants.

### Data analysis

IBM SPSS Statistics Version 27 was used to run all analyses [45]. To measure levels of psychological distress in the context of our sample, we calculated frequencies and percentages for categorical sociodemographic characteristics, and DASS scores by severity level for each wave. To measure psychological distress scores in the context of our sample, we calculated means and standard deviations for variables such as age and DASS total and subscale scores for each wave and disaggregated for males and females. To compare psychological distress across the two waves, we ran paired-samples t-tests to assess changes in DASS scores among the total sample and by sex from Wave 1 and Wave 2 (test-retest reliability for this sample: Cronbach’s alpha α = .74 for depression; α = .71 for anxiety; α = .72 for stress). Effect sizes were calculated using the standardized mean difference, *d*, for comparison between waves using an effect size coding calculator [46, 47]. Finally, to further analyze relationships between psychological distress and characteristics of our sample, for Wave 2 data only, we ran one-way ANOVA tests to assess differences in DASS scores across the sociodemographic characteristics including sex, age category, civil status, occupation, and income; we ran post hoc analyses with the Bonferroni correction to reduce type 1 error rate. Results were considered significant at the *p* < .05 level.

## Results

### Sociodemographic characteristics

Our sample of 1,956 Spanish adults consisted of about half males (51.2%, n = 1,002) with a mean age of 50.1 (SD = 14). Participants’ ages ranged from 20–90 years old, with the majority between 35–60 (54.9%, n = 1,073). Our sample was mostly married (61%, n = 1,193), employed in the private sector (35.1%, n = 686) or retired (20.4%, n = 399), and earned a household income between 1,000–1,999 euros per month (30.3%, n = 592). For a complete overview of sample characteristics, please see Table 1.

**Table 1:** Demographic characteristics of the sample (N = 1,956).

Variable	N(%)
**Age**	
*18–34*	339(17.3)
*35–60*	1073(54.9)
*61+*	544(27.8)
**Sex**	
*Male*	1002(51.2)
*Female*	911(46.6)
**Civil Status**	
*Single*	530(27.1)
*Married*	1193(61.0)
*Widowed*	58(3.0)
*Divorced or Separated*	175(8.9)
**Occupational Status**	
*Public sector*	365(18.7)
*Private sector*	686(35.1)
*Entrepreneur*	112(5.7)
*On leave*	31(1.6)
*Not working*	216(11.0)
*Retired*	399(20.4)
*Student*	23(1.2)
*Homemaker*	73(3.7
*Permanent incapacity for work*	35(1.8)
*Other economic inactivity*	16(0.8)
**Income**	
*Less than 1*,*000*	194(9.9)
*1*,*000–1*,*9999*	592(30.3)
*2*,*000–2*,*999*	419(21.4)
*3*,*000–3*,*999*	215(11.0)
*4*,*000–4*,*999*	85(4.3)
*5*,*000+*	38(1.9)
*Prefer not to say*	413(21.1)

### Mental health overview

Overall, the majority of participants had depression, anxiety, and stress levels in the normal category across both Waves (Table 2). However, a large portion of participants did report moderate to extremely severe depression (Wave 1: 561, 28.7%; Wave 2: 425, 21.7%), anxiety (Wave 1: 475, 24.3%; Wave 2: 379, 19.4%), and stress (Wave 1: 217, 11.1%; Wave 2: 250, 12.8%). Across mild to extremely severe depression and anxiety levels, all decreased from Wave 1 to Wave 2. Alternatively, the portion of participants who experienced severe and extremely severe stress levels increased from Wave 1 to Wave 2. Severe stress increased from 63 (3.2%) to 92 (4.7%) participants, and extremely severe stress increased from 17 (0.9%) to 26 (1.3%) participants.

**Table 2:** Frequencies and percentages of DASS levels by Wave.

Variable	Level	N (%)	
		Wave 1	Wave 2
**Depression**	*Normal*	1160(58.3)	1341(68.6)
	*Mild*	235(12.0)	190(9.7)
	*Moderate*	276(14.1)	215(11.0)
	*Severe*	143(7.3)	102(5.2)
	*Extremely Severe*	142(7.3)	108(5.5)
**Anxiety**	*Normal*	1349(69.0)	1468(75.1)
	*Mild*	132(6.7)	109(5.6)
	*Moderate*	229(11.7)	173(8.8)
	*Severe*	82(4.2)	60(3.1)
	*Extremely Severe*	164(8.4)	146(7.5)
**Stress**	*Normal*	1589(81.2)	1570(80.3)
	*Mild*	150(7.7)	136(7.0)
	*Moderate*	137(7.0)	132(6.7)
	*Severe*	63(3.2)	92(4.7)
	*Extremely Severe*	17(0.9)	26(1.3)

**Fig 1:**
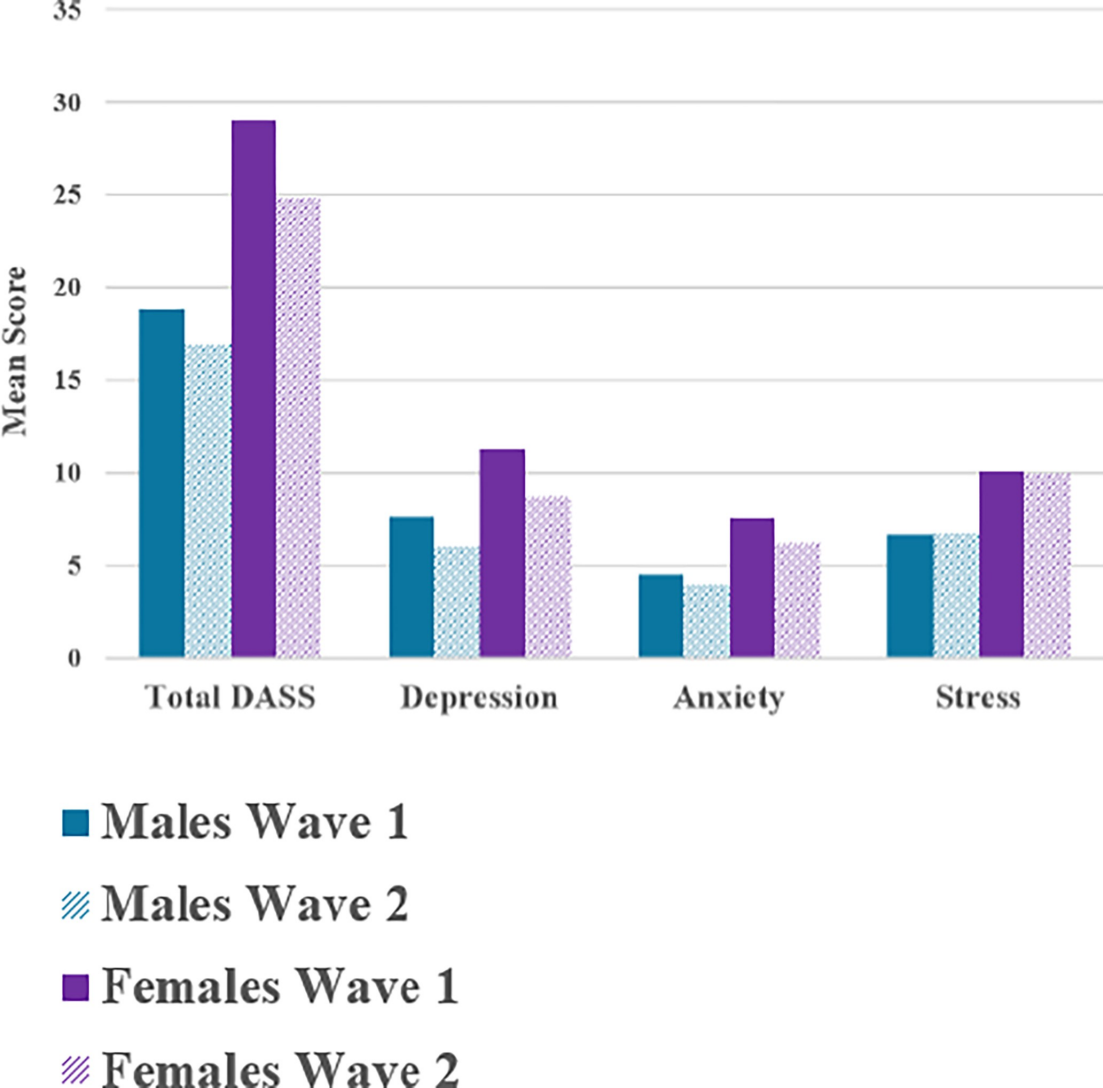
Mean DASS scores by sex across waves.

For DASS individual subscale scores and total scores, a similar pattern was found, where the depression, anxiety, and stress subscale mean scores and the total DASS scores across the total sample decreased from Wave 1 to Wave 2, even if very slightly (Table 3; Fig 1). Paired samples t-tests revealed these differences were statistically significant for depression (*t* = 10.224, *p* < .001), anxiety (*t* = 5.705, *p* < .001), and total DASS (*t* = 6.292, *p* < .001). Males’ and females’ depression, anxiety, and total DASS scores statistically significantly decreased from Wave 1 to Wave 2. Notably, stress scores remained approximately the same from Wave 1 to Wave 2, showing marginal decrease (lower scores) among the total sample and females, and a slight increase (higher scores) for males–not statistically significantly. Females exhibited a larger decrease in depression, anxiety, and total DASS scores compared to males from Wave 1 to Wave 2, though their improved score from Wave 2 remained higher than those of males, even with respect to scores reported in Wave 1. Effect size calculations showed a medium effect of depression scores being higher in Wave 1 than Wave 2 across the total sample (*d* = -0.203, 95% CI: -0.25, -0.16), and among females (*d* = -0.245, 95% CI: -0.31, -0.18); with a slightly smaller effect among males (*d* = -0.168, 95% CI: -0.23, -0.11). Overall, females evidenced much larger effect sizes for changes in anxiety and total DASS scores compared to males (Table 3). Changes in stress scores showed very minimal effects for both males and females, although increased for males (*d* = 0.008, 95% CI: -0.05, 0.07) and decreased for females (*d* = -0.012, 95% CI: -0.08, -0.05), all non-significantly.

**Table 3:** Change in depression, anxiety, and stress scores from the DASS-21 from April 2021 (Wave 1) to August 2022 (Wave 2) by sex.

Variable	*Total*		*t*	*d (95% CI)*	*Males*		*t*	*d (95% CI)*	*Females*		*t*	*d (95% CI)*
	Mean (SD)				Mean (SD)				Mean (SD)			
	Wave 1	Wave 2			Wave 1	Wave 2			Wave 1	Wave 2		
**Depression**	9.38(10.04)	7.34(9.29)	10.224**	**-0.203 (-0.25,-0.16)**	7.63(9.11)	6.1(8.57)	6.195**	**-0.168 (-0.23,-0.11)**	11.33(10.66)	8.72(9.86)	8.184**	**-0.245 (-0.31, -0.18)**
**Anxiety**	5.98(7.90)	5.05(7.32)	5.705**	**-0.118 (-0.16, -0.07)**	4.56(6.93)	4.04(6.71)	2.426*	**-0.075 (-0.14, -0.01)**	7.58(8.58)	6.18(7.8)	5.569**	**-0.163 (-0.23, -0.1)**
**Stress**	8.29(7.95)	8.28(8.72)	0.093	-0.001 (-0.05, 0.04)	6.67(7.17)	6.73(8.22)	-0.289	0.008 (-0.05, 0.07)	10.1(8.37)	10(8.95)	0.401	- 0.012 (-0.08, 0.05)
**Total DASS**	23.65(23.93)	20.66(23.52)	6.292**	**-0.125 (-0.17, -0.08)**	18.87(21.39)	16.88(21.7)	3.261**	**-0.094 (-0.15, -0.03)**	29.01(25.44)	24.89(24.74)	5.559**	**-0.162 (-0.23, -0.1)**

* signifies significance at the *p* < .05 level

** signifies significance at the p < .001 level

Total Sample N = 1956; males n = 1033; females n = 923

Note: a higher mean score of each DASS category and total represents worse experiences of the corresponding mental health variable.

### Mental health associations

One-way ANOVA analyses compared mean depression, anxiety, and stress scores from Wave 2 across sociodemographic characteristics (Table 4). Mean scores for depression, anxiety, and stress were statistically significantly different between males and females, where females had overall higher mean scores (depression: F(1,1954) = 39.671, *p* < .001; anxiety: F(1,1954) = 42.287, *p* < .001; stress: F(1,1954) = 70.417, *p* < .001). Similarly, mean scores for depression, anxiety, and stress were statistically significantly different depending on age (depression: F(2,1953) = 44.742, *p* < .001; anxiety: F(2,1953) = 35.675, *p* < .001; stress: F(2,1953) = 66.528, *p* < .001). Post hoc comparisons showed statistically significant differences in mean depression, anxiety, and stress scores across all age groups (*p* < .001), where participants aged 18–34 had the highest mean scores. Also, statistically significantly differences was found by type of occupation (depression: F(9,1946) = 8.042, *p* < .001; anxiety: F(9,1946) = 5.572, *p* < .001; stress: F(9,1946) = 9.191, *p* < .001). Thus, statistically significant differences were found for depression, anxiety, and stress between students and those who were retired (*p* < .001), being students who had the highest mean scores in the three measures of psychological distress; between working in the public sector and being a student for depression (*p* = .045); and between working in the public (*p* = .029) or private (*p* = .018) sector and being a student for anxiety (S1–S3 Tables).

**Table 4:** Associations between demographic characteristics and depression, anxiety, and stress scores in Wave 2 via one-way ANOVA (N = 1,956).

Variable	Depression			Anxiety			Stress		
	N (%)	Mean (SD)	F	N (%)	Mean (SD)	F	N (%)	Mean (SD)	F
Sex			39.671*			42.287*			70.417*
*Male*	1033(52.8)	6.10(8.57)		1033(52.8)	4.04(6.71)		1033(52.8)	6.74(8.22)	
*Female*	923(47.2)	8.73(9.86)		923(47.2)	6.18(7.8)		923(47.2)	9.99(8.95)	
Age Category			44.742*			35.675*			66.528*
*18–34*	339(17.3)	10.76(10.51)		339(17.3)	7.61(8.24)		339(17.3)	12.01(9.27)	
*35–60*	1073(54.9)	7.52(9.45)		1073(54.9)	5.07(7.5)		1073(54.9)	8.58(8.73)	
*61+*	544(27.8)	4.84(7.23)		544(27.8)	3.41(5.7)		544(27.8)	5.35(7.24)	
Civil Status			15.009*			6.014*			9.794*
*Single*	530(27.1)	9.56(10.05)		530(27.1)	6.06(7.69)		530(27.1)	10.0(9.08)	
*Married or Partnered*	1193(61.0)	6.39(8.82)		1193(61.0)	4.55(7.05)		1193(61)	7.69(8.55)	
*Widowed*	58(3.0)	8.28(10.06)		58(3.0)	6.45(8.69)		58(3.0)	7.83(9.29)	
*Divorced or Legally Separated*	175(8.9)	6.74(8.66)		175(8.9)	4.90(7.16)		175(8.9)	7.18(7.89)	
Occupation			8.042*			5.572*			9.191*
*Public sector*	365(18.7)	7.09(9.38)		365(18.7)	5.19(7.60)		365(18.7)	8.50(8.85)	
*Private sector*	686(35.1)	7.77(9.04)		686(35.1)	5.08(7.20)		686(35.1)	9.09(8.70)	
*Entrepreneur*	112(5.7)	7.16(9.31)		112(5.7)	5.48(8.26)		112(5.7)	9.57(10.06)	
*On leave*	31(1.6)	8.00(9.31)		31(1.6)	5.16(7.67)		31(1.6)	9.87(.28)	
*Not working*	216(11.0)	9.77(10.95)		216(11.0)	6.17(7.92)		216(11.0)	9.16(8.94)	
*Retired*	399(20.4)	4.51(7.04)		399(20.4)	3.28(5.54)		399(20.4)	5.02(7.04)	
*Student*	23(1.2)	13.57(13.14)		23(1.2)	10.52(10.22)		23(1.2)	13.83(10.07)	
*Homemaker*	73(3.7)	9.26(10.73)		73(3.7	6.3(7.18)		73(3.7)	9.81(8.74)	
*Permanent work incapacity*	35(1.8)	9.83(10.85)		35(1.8)	7.31(9.98)		35(1.8)	8.40(9.12)	
*Other economic inactivity*	16(0.8)	9.00(7.76)		16(0.8)	7.50(8.50)		16(0.8)	10.13(9.92)	
Income			7.012*			5.406*			3.534**
*Less than 1*,*000*	194(9.9)	10.39(10.6)		194(9.9)	7.65(8.82)		194(9.9)	10.53(10.08)	
*1*,*000–1*,*9999*	592(30.3)	7.84(9.22)		592(30.3)	5.14(7.16)		592(30.3)	8.47(8.57)	
*2*,*000–2*,*999*	419(21.4)	7.01(8.96)		419(21.4)	4.80(6.83)		419(21.4)	8.26(8.26)	
*3*,*000–3*,*999*	215(11.0)	5.57(7.73)		215(11.0)	4.38(7.02		215(11.0)	7.55(8.29)	
*4*,*000–4*,*999*	85(4.3)	4.21(7.26)		85(4.3)	3.62(6.95)		85(4.3)	6.21(8.21)	
*5*,*000+*	38(1.9)	6.21(7.93)		38(1.9)	3.84(6.48)		38(1.9)	7.12(7.66)	
*Prefer not to say*	413(21.1)	7.17(9.88)		413(21.1)	4.71(7.31)		413(21.1)	7.86(8.96)	

*p < .001

**p = .002

Note: a higher mean score of each DASS category and total represents worse experiences of the corresponding mental health variable.

There were also statistically significant differences between groups based on civil status (depression: F(3,1952) = 15.009, *p* < .001; anxiety: F(3,1952) = 6.014, *p* < .001; stress: F(3,1952) = 9.794, *p* < .001), where post hoc analyses revealed statistically significant differences between being single and married for depression, anxiety, and stress (*p* < .001); and for being single and divorced or separated for depression (*p* = .003) and stress (*p* < .001). It should be noted that depression and anxiety mean scores were highest among single and widowed participants, though stress was highest for single participants. Lastly, differences between groups based on income were statistically significant for all subscales (depression: F(6,1949) = 7.012, *p* < .001; anxiety: F(6,1949) = 5.406, *p* < .001; stress: F(6,1949) = 3.534, *p* = .002). The mean scores for depression, anxiety, and stress were highest among those who make less than 1,000 euros per month in their household and decreased slightly per each increasing income category; however, participants who made more than 5,000 euros per month in their household saw slight increases across depression and stress subscales. While several post hoc comparisons revealed statistically significant differences between several income levels, the post hoc comparisons did not show a statistically significant difference in mean scores for any subscale between the lowest and highest income categories (S1–S3 Tables).

## Discussion

Our data from 1,956 Spanish adults found that levels of psychological distress (i.e., depression, anxiety, and stress as subscales and a total DASS score) of the total sample in the context of the pandemic had improved slightly (i.e., scores lowered) from April 2021 to August 2022. However, as psychological distress among the sample improved, certain populations were experiencing worse psychological distress than others. For example, psychological distress was the poorest among females, and those who are either single or widowed. Psychological distress was also the poorest among those aged 18–34, and students. Additionally, psychological distress differed between income levels; although depression, anxiety, and stress were most exacerbated for those with the lowest income. Finally, while depression and anxiety saw larger improvements from Wave 1 to Wave 2, stress stayed about the same, and even increased slightly for males.

One of our most prominent findings was the notable disparity in psychological distress between males and females in our sample. We found that females experienced significantly higher levels of depression, anxiety, and stress compared to males. Early pandemic data found that females reported worse pandemic-related psychological distress compared to males, and our data shows that this trend persists two and a half years into the pandemic [6–8, 25, 27–31]. Further, all of females’ psychological distress scores were higher than males’, and while males saw less of an improvement in depression and anxiety from Wave 1 to Wave 2, females’ lowered scores at Wave 2 are still higher than males’ highest scores from Wave 1. The scientific literature points to some explanations for these findings related to childcare and occupational responsibilities.

One potential explanation for this mental health disparity between males and females can be attributed to *parental burnout*, which is defined by Melnyk and Lusk (2022) as when chronic stress and exhaustion occur and overwhelm a parent’s ability to cope and function, as a result of parents feeling physically, mentally, and emotionally exhausted. Early data on parental burnout during the pandemic found that levels of burnout were high, especially among females [9, 48–51]. Further, working parents were particularly at risk for experiencing parental burnout due to juggling demands and stressors both at work and in the home [52]. It is clear why the COVID-19 pandemic could exacerbate these stressors during lockdown periods and periods of strict quarantine regulations; though now that life has returned to a semblance of ‘normalcy’, it could be hypothesized that levels of parental burnout would decrease as well. However, our data and other data show that this is potentially not the case for females [53–58]. Additionally, several studies have found that parental burnout was worse among younger parents which could be contributing to our data that younger adults aged 18–34 are experiencing worse psychological distress than older adults [48, 51, 59–61].

Our data suggest that young adults aged 18–34 were suffering more psychological distress compared to middle aged and older adults. This is consistent with our data from April 2020 [17, 25], and data from other research both in Spain and globally [5, 32]. For example, one study of young adults aged 18–35 in the North of Spain found that one and a half years into the pandemic, young adults were suffering higher levels of depression, anxiety, and stress compared to the beginning of the pandemic, where levels were already elevated [5]. Our data also suggest that young adults who were students had more psychological distress as well, which is also consistent with research regarding the harsh impact that the pandemic had on college students’ lifestyles and mental health [3, 4, 62]. Particularly, the study by Jones et al. (2022) assessed college students during three waves of the pandemic and found that students were primarily resilient, but trends of worsening mental health suggest a possibility of delayed impact. Our data shows that students’ psychological distress are still at severe levels two and a half years into the pandemic, and suggests that more intervention or mental health resources are urgently needed for this young adult population who may be experiencing prolonged mental health struggles as a result of the pandemic.

Our research also found that participants with the lowest income tended to report greater symptoms of depression, anxiety, and stress than those with higher income. This result coincides with previous research from a cross-sectional epidemiological study carried out recently in Australia where the prevalence of mental disorders varied significantly across socioeconomic groups, with the lowest socioeconomic group exhibiting the highest prevalence of anxiety and other mental disorders [63]. This study found profound differences between the lower income participants and the higher income participants–a finding inconsistent with our own data. These results reinforce the need to develop interventions aimed at preserving the mental health of groups that are vulnerable due to low socioeconomic status.

Interestingly, our data suggest that while depression and anxiety symptoms tended to improve across the total sample, males, and females from Wave 1 to Wave 2; the same trend was not observed for stress. When the depression, anxiety, and stress scores were divided into levels of severity, the number of participants who experienced severe and extremely severe stress levels in Wave 1 increased by Wave 2. Further, the mean stress scores of the total sample only changed by 0.01 from Wave 1 to Wave 2, and 0.1 among females which are extremely marginal decrease. Contrarily, depression and anxiety scores decreased more drastically. For males, stress scores were found to have increased from Wave 1 to Wave 2. There is limited empirical literature regarding males’ stress in the context of the pandemic, most in relation to coping strategies [64, 65]. A study of Spanish adults from Catalonia in July 2021 associated stress levels with coping strategies among females versus males, though found that approximately 30% of males in the sample experienced moderate, severe, or extremely severe stress [65]; whereas our April 2021 data found 11% of our sample reported those levels of stress; 13% in August 2022.

While this data is, to the best of our knowledge, the first study that provides mental health data from two and a half years into the COVID-19 pandemic, it does have limitations. First, we do not have data among this sample prior to the pandemic, so we do not have context to participants’ mental health prior to the pandemic for a more thorough comparison. Second, our Wave 1 sample size was larger at 3,500 participants, and due to summer vacations in Spain, it was increasingly difficult to resurvey all of these participants in Wave 2, so we can only draw longitudinal conclusions among 1,956 of those participants who participated in both Wave 1 and Wave 2. This second limitation does not limit the interpretation of the current findings, though may miss valuable data and insight into the participants’ psychological distress that were not able to be resampled. Finally, although Netquest has a large sample of panelists across Spain and globally to recruit from and assures possibility of a representative sample, the nature of only recruiting through their panelists may limit generalizability of the findings to individuals who are not part of the Netquest panel.

Despite these limitations, this study has notable strengths. As stated above, this data is novel, as it is (to the best of our knowledge) the only research that has collected longitudinal data on a representative, country-wide sample of adults since the start of the COVID-19 pandemic into late 2022. We can draw conclusions of change between Wave 1 in April 2021 to Wave 2 in August 2022, making this data the first to provide a status update on the psychological distress of a representative sample of Spanish adults this far into the pandemic.

## Conclusions

This longitudinal survey study sought to assess the psychological distress (i.e., depression, anxiety, and stress) of adults in Spain during the COVID-19 pandemic and compare data between April 2021 and August 2022. Notably, we found that overall, psychological distress improved slightly from April 2021 to August 2022, though some populations were identified as more vulnerable than others. Females and young adults aged 18–34 –particularly students–continue to suffer more psychological distress compared to males and older adults. Further, low income exacerbated depression, anxiety, and stress; and males’ stress worsened over time. Our data provides an overview of the mental health of Spanish adults two and a half years into the pandemic, and provides novel evidence that even though life has resumed a sense of normalcy in regards to the COVID-19 pandemic, the mental health of key populations (e.g., females, young adults, students, low-income) is still suffering and further intervention and resources remain urgently needed. As our data suggests the pandemic effects on psychological distress persist two and a half years after the start of the pandemic, and are exacerbated among some risk populations, continued data collection on mental health is imperative as the world continues to manage this new normal, and global conflicts continue to mount with the potential of further exacerbating mental health. Future research should specifically explore why females and young adult populations are suffering from worse mental health with a focus on parents to inform targeted interventions; and attempt to identify the risk factors influencing the elevated stress levels over time, particularly for males to inform targeted interventions.

## Supporting information

S1 Table Bonferroni posthoc comparisons of total depression scores of the sample and demographic characteristics. (DOCX)

S2 Table Bonferroni posthoc comparisons of total anxiety scores of the sample and demographic characteristics. (DOCX)

S3 Table Bonferroni posthoc comparisons of total stress scores of the sample and demographic characteristics. (DOCX)

S1 Dataset (XLSX)
