# Supplementary material for: Our lifestyles are back to ‘normal’, but is our mental health? Longitudinal assessment of psychological distress during the COVID-19 pandemic among Spanish adults: April 2021 to August 2022
Source: PLOS Glob Public Health. 2024 Jul 17;4(7):e0003389. doi: 10.1371/journal.pgph.0003389 (PMC11253917; doi:10.1371/journal.pgph.0003389)
Supplement: S1 Table — (DOCX) [file pgph.0003389.s001.docx]

| **S1 Table.** Bonferroni posthoc comparisons of total depression scores of the sample and demographic characteristics. | | | | | |
| --- | --- | --- | --- | --- | --- |
| **Main Variable** | **Variable level 1** | **Variable level 2** | **Mean Difference** | **95% CI** | |
|  |  |  |  | **Lower Bound** | **Upper Bound** |
| **Age** | 18-34 | 35-60 | 3.230 | **1.873** | **4.588** |
|  |  | 61+ | 5.917 | **4.410** | **7.424** |
|  | 35-60 | 18-34 | -3.230 | **-4.588** | **-1.873** |
|  |  | 61+ | 2.686 | **1.540** | **3.833** |
|  | 61+ | 18-34 | -5.917 | **-7.424** | **-4.410** |
|  |  | 35-60 | -2.686 | **-3.833** | **-1.540** |
| **Civil Status** | Single | Married | 3.172 | **1.904** | **4.439** |
|  |  | Widowed | 1.286 | -2.072 | 4.645 |
|  |  | Divorced or Separated | 2.819 | **0.703** | **4.936** |
|  | Married | Single | -3.172 | **-4.439** | **-1.904** |
|  |  | Widowed | -1.885 | -5.150 | 1.380 |
|  |  | Divorced or Separated | -0.352 | -2.318 | 1.613 |
|  | Widowed | Single | -1.286 | -4.645 | 2.072 |
|  |  | Married | 1.885 | -1.380 | 5.150 |
|  |  | Divorced or Separated | 1.533 | -2.146 | 5.212 |
|  | Divorced or Separated | Single | -2.819 | **-4.936** | **-0.703** |
|  |  | Married | 0.352 | -1.613 | 2.318 |
|  |  | Widowed | -1.533 | -5.212 | 2.146 |
| **Occupation** | Public Sector | Private Sector | -0.676 | -2.611 | 1.259 |
|  |  | Entrepreneur | -0.070 | -3.296 | 3.156 |
|  |  | On Leave | -0.910 | -6.497 | 4.677 |
|  |  | Not Working | -2.678 | -5.242 | -0.114 |
|  |  | Retired | 2.579 | **0.416** | **4.742** |
|  |  | Student | -6.475 | **-12.895** | **-0.055** |
|  |  | Homemaker | -2.170 | -5.999 | 1.659 |
|  |  | Permanent Incapacity for work | -2.738 | -8.023 | 2.546 |
|  |  | Other economic activity | -1.910 | -9.538 | 5.718 |
|  | Private Sector | Public Sector | 0.676 | -1.259 | 2.611 |
|  |  | Entrepreneur | 0.606 | -2.438 | 3.650 |
|  |  | On Leave | -0.233 | -5.717 | 5.250 |
|  |  | Not Working | -2.002 | -4.332 | 0.328 |
|  |  | Retired | 3.255 | **1.375** | **5.136** |
|  |  | Student | -5.798 | -12.129 | 0.532 |
|  |  | Homemaker | -1.494 | -5.170 | 2.183 |
|  |  | Permanent Incapacity for work | -2.062 | -7.237 | 3.113 |
|  |  | Other economic activity | -1.233 | -8.786 | 6.319 |
|  | Entrepreneur | Public Sector | 0.070 | -3.156 | 3.296 |
|  |  | Private Sector | -0.606 | -3.650 | 2.438 |
|  |  | On Leave | -0.839 | -6.900 | 5.222 |
|  |  | Not Working | -2.608 | -6.085 | 0.870 |
|  |  | Retired | 2.649 | -0.544 | 5.843 |
|  |  | Student | -6.405 | -13.241 | 0.432 |
|  |  | Homemaker | -2.100 | -6.592 | 2.393 |
|  |  | Permanent Incapacity for work | -2.668 | -8.451 | 3.115 |
|  |  | Other economic activity | -1.839 | -9.821 | 6.142 |
|  | On Leave | Public Sector | 0.910 | -4.677 | 6.497 |
|  |  | Private Sector | 0.233 | -5.250 | 5.717 |
|  |  | Entrepreneur | 0.839 | -5.222 | 6.900 |
|  |  | Not Working | -1.769 | -7.504 | 3.967 |
|  |  | Retired | 3.489 | -2.080 | 9.057 |
|  |  | Student | -5.565 | -13.784 | 2.654 |
|  |  | Homemaker | -1.260 | -7.662 | 5.142 |
|  |  | Permanent Incapacity for work | -1.829 | -9.194 | 5.537 |
|  |  | Other economic activity | -1.000 | -10.193 | 8.193 |
|  | Not Working | Public Sector | 2.678 | **0.114** | **5.242** |
|  |  | Private Sector | 2.002 | -0.328 | 4.332 |
|  |  | Entrepreneur | 2.608 | -0.870 | 6.085 |
|  |  | On Leave | 1.769 | -3.967 | 7.504 |
|  |  | Retired | 5.257 | **2.735** | **7.780** |
|  |  | Student | -3.797 | -10.347 | 2.754 |
|  |  | Homemaker | 0.508 | -3.535 | 4.551 |
|  |  | Permanent Incapacity for work | -0.060 | -5.502 | 5.382 |
|  |  | Other economic activity | 0.769 | -6.969 | 8.506 |
|  | Retired | Public Sector | -2.579 | **-4.742** | **-0.416** |
|  |  | Private Sector | -3.255 | **-5.136** | **-1.375** |
|  |  | Entrepreneur | -2.649 | -5.843 | 0.544 |
|  |  | On Leave | -3.489 | -9.057 | 2.080 |
|  |  | Not Working | -5.257 | **-7.780** | **-2.735** |
|  |  | Student | -9.054 | **-15.458** | **-2.650** |
|  |  | Homemaker | -4.749 | **-8.551** | **-0.947** |
|  |  | Permanent Incapacity for work | -5.317 | **-10.582** | **-0.053** |
|  |  | Other economic activity | -4.489 | -12.103 | 3.126 |
|  | Student | Public Sector | 6.475 | **0.055** | **12.895** |
|  |  | Private Sector | 5.798 | -0.532 | 12.129 |
|  |  | Entrepreneur | 6.405 | -0.432 | 13.241 |
|  |  | On Leave | 5.565 | -2.654 | 13.784 |
|  |  | Not Working | 3.797 | -2.754 | 10.347 |
|  |  | Retired | 9.054 | 2.650 | 15.458 |
|  |  | Homemaker | 4.305 | -2.836 | 11.446 |
|  |  | Permanent Incapacity for work | 3.737 | -4.280 | 11.753 |
|  |  | Other economic activity | 4.565 | -5.157 | 14.287 |
|  | Homemaker | Public Sector | 2.170 | -1.659 | 5.999 |
|  |  | Private Sector | 1.494 | -2.183 | 5.170 |
|  |  | Entrepreneur | 2.100 | -2.393 | 6.592 |
|  |  | On Leave | 1.260 | -5.142 | 7.662 |
|  |  | Not Working | -0.508 | -4.551 | 3.535 |
|  |  | Retired | 4.749 | **0.947** | **8.551** |
|  |  | Student | -4.305 | -11.446 | 2.836 |
|  |  | Permanent Incapacity for work | -0.568 | -6.708 | 5.572 |
|  |  | Other economic activity | 0.260 | -7.984 | 8.504 |
|  | Permanent Incapacity for work | Public Sector | 2.738 | -2.546 | 8.023 |
|  |  | Private Sector | 2.062 | -3.113 | 7.237 |
|  |  | Entrepreneur | 2.668 | -3.115 | 8.451 |
|  |  | On Leave | 1.829 | -5.537 | 9.194 |
|  |  | Not Working | 0.060 | -5.382 | 5.502 |
|  |  | Retired | 5.317 | **0.053** | **10.582** |
|  |  | Student | -3.737 | -11.753 | 4.280 |
|  |  | Homemaker | 0.568 | -5.572 | 6.708 |
|  |  | Other economic activity | 0.829 | -8.184 | 9.841 |
|  | Other economic activity | Public Sector | 1.910 | -5.718 | 9.538 |
|  |  | Private Sector | 1.233 | -6.319 | 8.786 |
|  |  | Entrepreneur | 1.839 | -6.142 | 9.821 |
|  |  | On Leave | 1.000 | -8.193 | 10.193 |
|  |  | Not Working | -0.769 | -8.506 | 6.969 |
|  |  | Retired | 4.489 | -3.126 | 12.103 |
|  |  | Student | -4.565 | -14.287 | 5.157 |
|  |  | Homemaker | -0.260 | -8.504 | 7.984 |
|  |  | Permanent Incapacity for work | -0.829 | -9.841 | 8.184 |
| **Income** | Less than 1,000 | 1,000-1,9999 | 2.547 | **0.230** | **4.864** |
|  |  | 2,000-2,999 | 3.380 | **0.947** | **5.812** |
|  |  | 3,000-3,999 | 4.820 | **2.046** | **7.593** |
|  |  | 4,000-4,999 | 6.180 | **2.537** | **9.823** |
|  |  | 5,000+ | 4.181 | -0.788 | 9.150 |
|  | 1,000-1,999 | Less than 1,000 | -2.547 | **-4.864** | **-0.230** |
|  |  | 2,000-2,9999 | 0.833 | -0.956 | 2.621 |
|  |  | 3,000-3,999 | 2.273 | **0.042** | **4.503** |
|  |  | 4,000-4,999 | 3.633 | **0.384** | **6.882** |
|  |  | 5,000+ | 1.634 | -3.053 | 6.321 |
|  | 2,000-2,999 | Less than 1,000 | -3.380 | **-5.812** | **-0.947** |
|  |  | 1,000-1,9999 | -0.833 | -2.621 | 0.956 |
|  |  | 3,000-3,999 | 1.440 | -0.910 | 3.790 |
|  |  | 4,000-4,999 | 2.800 | -0.532 | 6.132 |
|  |  | 5,000+ | 0.801 | -3.944 | 5.547 |
|  | 3,000-3,999 | Less than 1,000 | -4.820 | **-7.593** | **-2.046** |
|  |  | 1,000-1,9999 | -2.273 | **-4.503** | **-0.042** |
|  |  | 2,000-2,999 | -1.440 | -3.790 | 0.910 |
|  |  | 4,000-4,999 | 1.360 | -2.228 | 4.949 |
|  |  | 5,000+ | -0.638 | -5.567 | 4.291 |
|  | 4,000-4,999 | Less than 1,000 | -6.180 | **-9.823** | **-2.537** |
|  |  | 1,000-1,9999 | -3.633 | **-6.882** | **-0.384** |
|  |  | 2,000-2,999 | -2.800 | -6.132 | 0.532 |
|  |  | 3,000-3,999 | -1.360 | -4.949 | 2.228 |
|  |  | 5,000+ | -1.999 | -7.465 | 3.467 |
|  | 5,000+ | Less than 1,000 | -4.181 | -9.150 | 0.788 |
|  |  | 1,000-1,9999 | -1.634 | -6.321 | 3.053 |
|  |  | 2,000-2,999 | -0.801 | -5.547 | 3.944 |
|  |  | 3,000-3,999 | 0.638 | -4.291 | 5.567 |
|  |  | 4,000-4,999 | 1.999 | -3.467 | 7.465 |
|  |  |  |  |  |  |
