# Supplementary material for: Our lifestyles are back to ‘normal’, but is our mental health? Longitudinal assessment of psychological distress during the COVID-19 pandemic among Spanish adults: April 2021 to August 2022
Source: PLOS Glob Public Health. 2024 Jul 17;4(7):e0003389. doi: 10.1371/journal.pgph.0003389 (PMC11253917; doi:10.1371/journal.pgph.0003389)
Supplement: S2 Table — (DOCX) [file pgph.0003389.s002.docx]

| **S2 Table.** Bonferroni posthoc comparisons of total anxiety scores of the sample and demographic characteristics. | | | | | | | | | | | |
| --- | --- | --- | --- | --- | --- | --- | --- | --- | --- | --- | --- |
| **Main Variable** | | **Variable level 1** | | **Variable level 2** | | **Mean Difference** | | | **95% CI** | | |
|  |  |  |  |  |  |  |  |  | **Lower Bound** | | **Upper Bound** |
| **Age** | | 18-34 | | 35-60 | | 2.539 | | | **1.465** | | **3.612** |
|  |  |  |  | 61+ | | 4.203 | | | **3.010** | | **5.395** |
|  |  | 35-60 | | 18-34 | | -2.539 | | | **-3.612** | | **-1.465** |
|  |  |  |  | 61+ | | 1.664 | | | **0.757** | | **2.571** |
|  |  | 61+ | | 18-34 | | -4.203 | | | **-5.395** | | **-3.010** |
|  |  |  |  | 35-60 | | -1.664 | | | **-2.571** | | **-0.757** |
| **Civil Status** | | Single | | Married | | 1.513 | | | **0.508** | | **2.518** |
|  |  |  |  | Widowed | | -0.384 | | | -3.047 | | 2.279 |
|  |  |  |  | Divorced or Separated | | 1.161 | | | -0.517 | | 2.840 |
|  |  | Married | | Single | | -1.513 | | | **-2.518** | | **-0.508** |
|  |  |  |  | Widowed | | -1.897 | | | -4.485 | | 0.692 |
|  |  |  |  | Divorced or Separated | | -0.351 | | | -1.910 | | 1.207 |
|  |  | Widowed | | Single | | 0.384 | | | -2.279 | | 3.047 |
|  |  |  |  | Married | | 1.897 | | | -0.692 | | 4.485 |
|  |  |  |  | Divorced or Separated | | 1.545 | | | -1.371 | | 4.462 |
|  |  | Divorced or Separated | | Single | | -1.161 | | | -2.840 | | 0.517 |
|  |  |  |  | Married | | 0.351 | | | -1.207 | | 1.910 |
|  |  |  |  | Widowed | | -1.545 | | | -4.462 | | 1.371 |
| **Occupation** | | Public Sector | | Private Sector | | 0.110 | | | -1.422 | | 1.642 |
|  |  |  |  | Entrepreneur | | -0.288 | | | -2.842 | | 2.267 |
|  |  |  |  | On Leave | | 0.033 | | | -4.391 | | 4.458 |
|  |  |  |  | Not Working | | -0.972 | | | -3.002 | | 1.058 |
|  |  |  |  | Retired | | 1.916 | | | **0.203** | | **3.629** |
|  |  |  |  | Student | | -5.327 | | | -10.411 | | -0.243 |
|  |  |  |  | Homemaker | | -1.107 | | | -4.139 | | 1.925 |
|  |  |  |  | Permanent Incapacity for work | | -2.120 | | | -6.305 | | 2.065 |
|  |  |  |  | Other economic activity | | -2.305 | | | -8.346 | | 3.735 |
|  |  | Private Sector | | Public Sector | | -0.110 | | | -1.642 | | 1.422 |
|  |  |  |  | Entrepreneur | | -0.398 | | | -2.808 | | 2.013 |
|  |  |  |  | On Leave | | -0.077 | | | -4.419 | | 4.266 |
|  |  |  |  | Not Working | | -1.082 | | | -2.927 | | 0.763 |
|  |  |  |  | Retired | | 1.806 | | | **0.317** | | **3.295** |
|  |  |  |  | Student | | -5.437 | | | **-10.450** | | **-0.424** |
|  |  |  |  | Homemaker | | -1.217 | | | -4.128 | | 1.695 |
|  |  |  |  | Permanent Incapacity for work | | -2.230 | | | -6.328 | | 1.868 |
|  |  |  |  | Other economic activity | | -2.415 | | | -8.396 | | 3.565 |
|  |  | Entrepreneur | | Public Sector | | 0.288 | | | -2.267 | | 2.842 |
|  |  |  |  | Private Sector | | 0.398 | | | -2.013 | | 2.808 |
|  |  |  |  | On Leave | | 0.321 | | | -4.479 | | 5.120 |
|  |  |  |  | Not Working | | -0.685 | | | -3.438 | | 2.069 |
|  |  |  |  | Retired | | 2.204 | | | -0.325 | | 4.733 |
|  |  |  |  | Student | | -5.040 | | | -10.454 | | 0.374 |
|  |  |  |  | Homemaker | | -0.819 | | | -4.377 | | 2.738 |
|  |  |  |  | Permanent Incapacity for work | | -1.832 | | | -6.412 | | 2.748 |
|  |  |  |  | Other economic activity | | -2.018 | | | -8.338 | | 4.303 |
|  |  | On Leave | | Public Sector | | -0.033 | | | -4.458 | | 4.391 |
|  |  |  |  | Private Sector | | 0.077 | | | -4.266 | | 4.419 |
|  |  |  |  | Entrepreneur | | -0.321 | | | -5.120 | | 4.479 |
|  |  |  |  | Not Working | | -1.005 | | | -5.548 | | 3.537 |
|  |  |  |  | Retired | | 1.883 | | | -2.526 | | 6.293 |
|  |  |  |  | Student | | -5.360 | | | -11.869 | | 1.148 |
|  |  |  |  | Homemaker | | -1.140 | | | -6.210 | | 3.930 |
|  |  |  |  | Permanent Incapacity for work | | -2.153 | | | -7.986 | | 3.680 |
|  |  |  |  | Other economic activity | | -2.339 | | | -9.619 | | 4.941 |
|  |  | Not Working | | Public Sector | | 0.972 | | | -1.058 | | 3.002 |
|  |  |  |  | Private Sector | | 1.082 | | | -0.763 | | 2.927 |
|  |  |  |  | Entrepreneur | | 0.685 | | | -2.069 | | 3.438 |
|  |  |  |  | On Leave | | 1.005 | | | -3.537 | | 5.548 |
|  |  |  |  | Retired | | 2.888 | | | **0.891** | | **4.886** |
|  |  |  |  | Student | | -4.355 | | | -9.542 | | 0.832 |
|  |  |  |  | Homemaker | | -0.135 | | | -3.336 | | 3.067 |
|  |  |  |  | Permanent Incapacity for work | | -1.148 | | | -5.457 | | 3.162 |
|  |  |  |  | Other economic activity | | -1.333 | | | -7.461 | | 4.794 |
|  |  | Retired | | Public Sector | | -1.916 | | | **-3.629** | | **-0.203** |
|  |  |  |  | Private Sector | | -1.806 | | | **-3.295** | | **-0.317** |
|  |  |  |  | Entrepreneur | | -2.204 | | | -4.733 | | 0.325 |
|  |  |  |  | On Leave | | -1.883 | | | -6.293 | | 2.526 |
|  |  |  |  | Not Working | | -2.888 | | | **-4.886** | | **-0.891** |
|  |  |  |  | Student | | -7.244 | | | **-12.315** | | **-2.172** |
|  |  |  |  | Homemaker | | -3.023 | | | -6.034 | | -0.013 |
|  |  |  |  | Permanent Incapacity for work | | -4.036 | | | -8.205 | | 0.133 |
|  |  |  |  | Other economic activity | | -4.222 | | | -10.252 | | 1.808 |
|  |  | Student | | Public Sector | | 5.327 | | | **0.243** | | **10.411** |
|  |  |  |  | Private Sector | | 5.437 | | | **0.424** | | **10.450** |
|  |  |  |  | Entrepreneur | | 5.040 | | | -0.374 | | 10.454 |
|  |  |  |  | On Leave | | 5.360 | | | -1.148 | | 11.869 |
|  |  |  |  | Not Working | | 4.355 | | | -0.832 | | 9.542 |
|  |  |  |  | Retired | | 7.244 | | | **2.172** | | **12.315** |
|  |  |  |  | Homemaker | | 4.220 | | | -1.435 | | 9.875 |
|  |  |  |  | Permanent Incapacity for work | | 3.207 | | | -3.141 | | 9.555 |
|  |  |  |  | Other economic activity | | 3.022 | | | -4.677 | | 10.721 |
|  |  | Homemaker | | Public Sector | | 1.107 | | | -1.925 | | 4.139 |
|  |  |  |  | Private Sector | | 1.217 | | | -1.695 | | 4.128 |
|  |  |  |  | Entrepreneur | | 0.819 | | | -2.738 | | 4.377 |
|  |  |  |  | On Leave | | 1.140 | | | -3.930 | | 6.210 |
|  |  |  |  | Not Working | | 0.135 | | | -3.067 | | 3.336 |
|  |  |  |  | Retired | | 3.023 | | | **0.013** | | **6.034** |
|  |  |  |  | Student | | -4.220 | | | -9.875 | | 1.435 |
|  |  |  |  | Permanent Incapacity for work | | -1.013 | | | -5.875 | | 3.849 |
|  |  |  |  | Other economic activity | | -1.199 | | | -7.727 | | 5.330 |
|  |  | Permanent Incapacity for work | | Public Sector | | 2.120 | | | -2.065 | | 6.305 |
|  |  |  |  | Private Sector | | 2.230 | | | -1.868 | | 6.328 |
|  |  |  |  | Entrepreneur | | 1.832 | | | -2.748 | | 6.412 |
|  |  |  |  | On Leave | | 2.153 | | | -3.680 | | 7.986 |
|  |  |  |  | Not Working | | 1.148 | | | -3.162 | | 5.457 |
|  |  |  |  | Retired | | 4.036 | | | -0.133 | | 8.205 |
|  |  |  |  | Student | | -3.207 | | | -9.555 | | 3.141 |
|  |  |  |  | Homemaker | | 1.013 | | | -3.849 | | 5.875 |
|  |  |  |  | Other economic activity | | -0.186 | | | -7.323 | | 6.951 |
|  |  | Other economic activity | | Public Sector | | 2.305 | | | -3.735 | | 8.346 |
|  |  |  |  | Private Sector | | 2.415 | | | -3.565 | | 8.396 |
|  |  |  |  | Entrepreneur | | 2.018 | | | -4.303 | | 8.338 |
|  |  |  |  | On Leave | | 2.339 | | | -4.941 | | 9.619 |
|  |  |  |  | Not Working | | 1.333 | | | -4.794 | | 7.461 |
|  |  |  |  | Retired | | 4.222 | | | -1.808 | | 10.252 |
|  |  |  |  | Student | | -3.022 | | | -10.721 | | 4.677 |
|  |  |  |  | Homemaker | | 1.199 | | | -5.330 | | 7.727 |
|  |  |  |  | Permanent Incapacity for work | | 0.186 | | | -6.951 | | 7.323 |
| **Income** | | Less than 1,000 | | 1,000-1,9999 | | 2.514 | | | **0.685** | | **4.344** |
|  |  |  |  | 2,000-2,999 | | 2.852 | | | **0.932** | | **4.773** |
|  |  |  |  | 3,000-3,999 | | 3.268 | | | **1.079** | | **5.458** |
|  |  |  |  | 4,000-4,999 | | 4.026 | | | **1.150** | | **6.902** |
|  |  |  |  | 5,000+ | | 3.807 | | | -0.115 | | 7.730 |
|  |  | 1,000-1999 | | Less than 1,000 | | -2.514 | | | **-4.344** | | **-0.685** |
|  |  |  |  | 2,000-2,9999 | | 0.338 | | | -1.074 | | 1.750 |
|  |  |  |  | 3,000-3,999 | | 0.754 | | | -1.007 | | 2.514 |
|  |  |  |  | 4,000-4,999 | | 1.512 | | | -1.053 | | 4.076 |
|  |  |  |  | 5,000+ | | 1.293 | | | -2.407 | | 4.993 |
|  |  | 2,000-2,999 | | Less than 1,000 | | -2.852 | | | **-4.773** | | **-0.932** |
|  |  |  |  | 1,000-1,9999 | | -0.338 | | | -1.750 | | 1.074 |
|  |  |  |  | 3,000-3,999 | | 0.416 | | | -1.439 | | 2.271 |
|  |  |  |  | 4,000-4,999 | | 1.174 | | | -1.457 | | 3.804 |
|  |  |  |  | 5,000+ | | 0.955 | | | -2.791 | | 4.701 |
|  |  | 3,000-3,999 | | Less than 1,000 | | -3.268 | | | **-5.458** | | **-1.079** |
|  |  |  |  | 1,000-1,9999 | | -0.754 | | | -2.514 | | 1.007 |
|  |  |  |  | 2,000-2,999 | | -0.416 | | | -2.271 | | 1.439 |
|  |  |  |  | 4,000-4,999 | | 0.758 | | | -2.075 | | 3.591 |
|  |  |  |  | 5,000+ | | 0.539 | | | -3.352 | | 4.430 |
|  |  | 4,000-4,999 | | Less than 1,000 | | -4.026 | | | **-6.902** | | **-1.150** |
|  |  |  |  | 1,000-1,9999 | | -1.512 | | | -4.076 | | 1.053 |
|  |  |  |  | 2,000-2,999 | | -1.174 | | | -3.804 | | 1.457 |
|  |  |  |  | 3,000-3,999 | | -0.758 | | | -3.591 | | 2.075 |
|  |  |  |  | 5,000+ | | -0.219 | | | -4.534 | | 4.096 |
|  |  | 5,000+ | | Less than 1,000 | | -3.807 | | | -7.730 | | 0.115 |
|  |  |  |  | 1,000-1,9999 | | -1.293 | | | -4.993 | | 2.407 |
|  |  |  |  | 2,000-2,999 | | -0.955 | | | -4.701 | | 2.791 |
|  |  |  |  | 3,000-3,999 | | -0.539 | | | -4.430 | | 3.352 |
|  |  |  |  | 4,000-4,999 | | 0.219 | | | -4.096 | | 4.534 |
|  | |  | |  | |  | | |  | |  |
| **Appendix C.** Bonferroni posthoc comparisons of total stress scores of the sample and demographic characteristics. | | | | | | | | | |  |  |
| **Main Variable** | **Variable level 1** | | **Variable level 2** | | **Mean Difference** | | **95% CI** | | |  |  |
|  |  |  |  |  |  |  | **Lower Bound** | **Upper Bound** | |  |  |
| **Age** | 18-34 | | 35-60 | | 3.424 | | **2.164** | **4.685** | |  |  |
|  |  |  | 61+ | | 6.660 | | **5.260** | **8.060** | |  |  |
|  | 35-60 | | 18-34 | | -3.424 | | **-4.685** | **-2.164** | |  |  |
|  |  |  | 61+ | | 3.236 | | **2.171** | **4.301** | |  |  |
|  | 61+ | | 18-34 | | -6.660 | | **-8.060** | **-5.260** | |  |  |
|  |  |  | 35-60 | | -3.236 | | **-4.301** | **-2.171** | |  |  |
| **Civil Status** | Single | | Married | | 2.308 | | **1.114** | **3.503** | |  |  |
|  |  |  | Widowed | | 2.172 | | -0.992 | 5.337 | |  |  |
|  |  |  | Divorced or Separated | | 2.823 | | **0.828** | **4.818** | |  |  |
|  | Married | | Single | | -2.308 | | **-3.503** | **-1.114** | |  |  |
|  |  |  | Widowed | | -0.136 | | -3.213 | 2.941 | |  |  |
|  |  |  | Divorced or Separated | | 0.514 | | -1.338 | 2.367 | |  |  |
|  | Widowed | | Single | | -2.172 | | -5.337 | 0.992 | |  |  |
|  |  |  | Married | | 0.136 | | -2.941 | 3.213 | |  |  |
|  |  |  | Divorced or Separated | | 0.650 | | -2.817 | 4.117 | |  |  |
|  | Divorced or Separated | | Single | | -2.823 | | **-4.818** | **-0.828** | |  |  |
|  |  |  | Married | | -0.514 | | -2.367 | 1.338 | |  |  |
|  |  |  | Widowed | | -0.650 | | -4.117 | 2.817 | |  |  |
| **Occupation** | Public Sector | | Private Sector | | -0.597 | | -2.409 | 1.215 | |  |  |
|  |  |  | Entrepreneur | | -1.078 | | -4.099 | 1.942 | |  |  |
|  |  |  | On Leave | | -1.378 | | -6.609 | 3.854 | |  |  |
|  |  |  | Not Working | | -0.664 | | -3.065 | 1.736 | |  |  |
|  |  |  | Retired | | 3.471 | | **1.445** | **5.496** | |  |  |
|  |  |  | Student | | -5.333 | | -11.345 | 0.679 | |  |  |
|  |  |  | Homemaker | | -1.315 | | -4.900 | 2.270 | |  |  |
|  |  |  | Permanent Incapacity for work | | 0.093 | | -4.855 | 5.041 | |  |  |
|  |  |  | Other economic activity | | -1.632 | | -8.774 | 5.511 | |  |  |
|  | Private Sector | | Public Sector | | 0.597 | | -1.215 | 2.409 | |  |  |
|  |  |  | Entrepreneur | | -0.481 | | -3.331 | 2.369 | |  |  |
|  |  |  | On Leave | | -0.781 | | -5.915 | 4.354 | |  |  |
|  |  |  | Not Working | | -0.067 | | -2.249 | 2.115 | |  |  |
|  |  |  | Retired | | 4.068 | | **2.307** | **5.828** | |  |  |
|  |  |  | Student | | -4.736 | | -10.663 | 1.192 | |  |  |
|  |  |  | Homemaker | | -0.718 | | -4.160 | 2.725 | |  |  |
|  |  |  | Permanent Incapacity for work | | 0.690 | | -4.155 | 5.536 | |  |  |
|  |  |  | Other economic activity | | -1.035 | | -8.107 | 6.037 | |  |  |
|  | Entrepreneur | | Public Sector | | 1.078 | | -1.942 | 4.099 | |  |  |
|  |  |  | Private Sector | | 0.481 | | -2.369 | 3.331 | |  |  |
|  |  |  | On Leave | | -0.300 | | -5.975 | 5.376 | |  |  |
|  |  |  | Not Working | | 0.414 | | -2.842 | 3.670 | |  |  |
|  |  |  | Retired | | 4.549 | | **1.559** | **7.539** | |  |  |
|  |  |  | Student | | -4.255 | | -10.656 | 2.147 | |  |  |
|  |  |  | Homemaker | | -0.237 | | -4.443 | 3.970 | |  |  |
|  |  |  | Permanent Incapacity for work | | 1.171 | | -4.244 | 6.587 | |  |  |
|  |  |  | Other economic activity | | -0.554 | | -8.027 | 6.920 | |  |  |
|  | On Leave | | Public Sector | | 1.378 | | -3.854 | 6.609 | |  |  |
|  |  |  | Private Sector | | 0.781 | | -4.354 | 5.915 | |  |  |
|  |  |  | Entrepreneur | | 0.300 | | -5.376 | 5.975 | |  |  |
|  |  |  | Not Working | | 0.714 | | -4.657 | 6.084 | |  |  |
|  |  |  | Retired | | 4.848 | | -0.365 | 10.062 | |  |  |
|  |  |  | Student | | -3.955 | | -11.651 | 3.741 | |  |  |
|  |  |  | Homemaker | | 0.063 | | -5.932 | 6.057 | |  |  |
|  |  |  | Permanent Incapacity for work | | 1.471 | | -5.426 | 8.368 | |  |  |
|  |  |  | Other economic activity | | -0.254 | | -8.862 | 8.354 | |  |  |
|  | Not Working | | Public Sector | | 0.664 | | -1.736 | 3.065 | |  |  |
|  |  |  | Private Sector | | 0.067 | | -2.115 | 2.249 | |  |  |
|  |  |  | Entrepreneur | | -0.414 | | -3.670 | 2.842 | |  |  |
|  |  |  | On Leave | | -0.714 | | -6.084 | 4.657 | |  |  |
|  |  |  | Retired | | 4.135 | | **1.773** | **6.497** | |  |  |
|  |  |  | Student | | -4.669 | | -10.802 | 1.465 | |  |  |
|  |  |  | Homemaker | | -0.651 | | -4.437 | 3.135 | |  |  |
|  |  |  | Permanent Incapacity for work | | 0.757 | | -4.338 | 5.853 | |  |  |
|  |  |  | Other economic activity | | -0.968 | | -8.213 | 6.278 | |  |  |
|  | Retired | | Public Sector | | -3.471 | | **-5.496** | **-1.445** | |  |  |
|  |  |  | Private Sector | | -4.068 | | **-5.828** | **-2.307** | |  |  |
|  |  |  | Entrepreneur | | -4.549 | | **-7.539** | **-1.559** | |  |  |
|  |  |  | On Leave | | -4.848 | | -10.062 | 0.365 | |  |  |
|  |  |  | Not Working | | -4.135 | | **-6.497** | **-1.773** | |  |  |
|  |  |  | Student | | -8.804 | | **-14.800** | **-2.807** | |  |  |
|  |  |  | Homemaker | | -4.786 | | **-8.345** | **-1.226** | |  |  |
|  |  |  | Permanent Incapacity for work | | -3.377 | | -8.307 | 1.552 | |  |  |
|  |  |  | Other economic activity | | -5.102 | | -12.232 | 2.027 | |  |  |
|  | Student | | Public Sector | | 5.333 | | -0.679 | 11.345 | |  |  |
|  |  |  | Private Sector | | 4.736 | | -1.192 | 10.663 | |  |  |
|  |  |  | Entrepreneur | | 4.255 | | -2.147 | 10.656 | |  |  |
|  |  |  | On Leave | | 3.955 | | -3.741 | 11.651 | |  |  |
|  |  |  | Not Working | | 4.669 | | -1.465 | 10.802 | |  |  |
|  |  |  | Retired | | 8.804 | | **2.807** | **14.800** | |  |  |
|  |  |  | Homemaker | | 4.018 | | -2.669 | 10.704 | |  |  |
|  |  |  | Permanent Incapacity for work | | 5.426 | | -2.080 | 12.932 | |  |  |
|  |  |  | Other economic activity | | 3.701 | | -5.402 | 12.804 | |  |  |
|  | Homemaker | | Public Sector | | 1.315 | | -2.270 | 4.900 | |  |  |
|  |  |  | Private Sector | | 0.718 | | -2.725 | 4.160 | |  |  |
|  |  |  | Entrepreneur | | 0.237 | | -3.970 | 4.443 | |  |  |
|  |  |  | On Leave | | -0.063 | | -6.057 | 5.932 | |  |  |
|  |  |  | Not Working | | 0.651 | | -3.135 | 4.437 | |  |  |
|  |  |  | Retired | | 4.786 | | **1.226** | **8.345** | |  |  |
|  |  |  | Student | | -4.018 | | -10.704 | 2.669 | |  |  |
|  |  |  | Permanent Incapacity for work | | 1.408 | | -4.341 | 7.157 | |  |  |
|  |  |  | Other economic activity | | -0.317 | | -8.036 | 7.402 | |  |  |
|  | Permanent Incapacity for work | | Public Sector | | -0.093 | | -5.041 | 4.855 | |  |  |
|  |  |  | Private Sector | | -0.690 | | -5.536 | 4.155 | |  |  |
|  |  |  | Entrepreneur | | -1.171 | | -6.587 | 4.244 | |  |  |
|  |  |  | On Leave | | -1.471 | | -8.368 | 5.426 | |  |  |
|  |  |  | Not Working | | -0.757 | | -5.853 | 4.338 | |  |  |
|  |  |  | Retired | | 3.377 | | -1.552 | 8.307 | |  |  |
|  |  |  | Student | | -5.426 | | -12.932 | 2.080 | |  |  |
|  |  |  | Homemaker | | -1.408 | | -7.157 | 4.341 | |  |  |
|  |  |  | Other economic activity | | -1.725 | | -10.164 | 6.714 | |  |  |
|  | Other economic activity | | Public Sector | | 1.632 | | -5.511 | 8.774 | |  |  |
|  |  |  | Private Sector | | 1.035 | | -6.037 | 8.107 | |  |  |
|  |  |  | Entrepreneur | | 0.554 | | -6.920 | 8.027 | |  |  |
|  |  |  | On Leave | | 0.254 | | -8.354 | 8.862 | |  |  |
|  |  |  | Not Working | | 0.968 | | -6.278 | 8.213 | |  |  |
|  |  |  | Retired | | 5.102 | | -2.027 | 12.232 | |  |  |
|  |  |  | Student | | -3.701 | | -12.804 | 5.402 | |  |  |
|  |  |  | Homemaker | | 0.317 | | -7.402 | 8.036 | |  |  |
|  |  |  | Permanent Incapacity for work | | 1.725 | | -6.714 | 10.164 | |  |  |
| **Income** | Less than 1,000 | | 1,000-1,9999 | | 2.056 | | -0.131 | 4.243 | |  |  |
|  |  |  | 2,000-2,999 | | 2.268 | | -0.027 | 4.564 | |  |  |
|  |  |  | 3,000-3,999 | | 2.972 | | **0.355** | **5.590*** | |  |  |
|  |  |  | 4,000-4,999 | | 4.314 | | **0.876** | **7.752*** | |  |  |
|  |  |  | 5,000+ | | 3.421 | | -1.269 | 8.110 | |  |  |
|  | 1,000-1,999 | | Less than 1,000 | | -2.056 | | -4.243 | 0.131 | |  |  |
|  |  |  | 2,000-2,9999 | | 0.212 | | -1.476 | 1.899 | |  |  |
|  |  |  | 3,000-3,999 | | 0.916 | | -1.189 | 3.021 | |  |  |
|  |  |  | 4,000-4,999 | | 2.258 | | -0.808 | 5.324 | |  |  |
|  |  |  | 5,000+ | | 1.364 | | -3.059 | 5.788 | |  |  |
|  | 2,000-2,999 | | Less than 1,000 | | -2.268 | | -4.564 | 0.027 | |  |  |
|  |  |  | 1,000-1,9999 | | -0.212 | | -1.899 | 1.476 | |  |  |
|  |  |  | 3,000-3,999 | | 0.704 | | -1.513 | 2.922 | |  |  |
|  |  |  | 4,000-4,999 | | 2.046 | | -1.098 | 5.190 | |  |  |
|  |  |  | 5,000+ | | 1.152 | | -3.326 | 5.631 | |  |  |
|  | 3,000-3,999 | | Less than 1,000 | | -2.972 | | **-5.590** | **-0.355*** | |  |  |
|  |  |  | 1,000-1,9999 | | -0.916 | | -3.021 | 1.189 | |  |  |
|  |  |  | 2,000-2,999 | | -0.704 | | -2.922 | 1.513 | |  |  |
|  |  |  | 4,000-4,999 | | 1.342 | | -2.045 | 4.728 | |  |  |
|  |  |  | 5,000+ | | 0.448 | | -4.203 | 5.100 | |  |  |
|  | 4,000-4,999 | | Less than 1,000 | | -4.314 | | **-7.752** | **-0.876*** | |  |  |
|  |  |  | 1,000-1,9999 | | -2.258 | | -5.324 | 0.808 | |  |  |
|  |  |  | 2,000-2,999 | | -2.046 | | -5.190 | 1.098 | |  |  |
|  |  |  | 3,000-3,999 | | -1.342 | | -4.728 | 2.045 | |  |  |
|  |  |  | 5,000+ | | -0.894 | | -6.052 | 4.265 | |  |  |
|  | 5,000+ | | Less than 1,000 | | -3.421 | | -8.110 | 1.269 | |  |  |
|  |  |  | 1,000-1,9999 | | -1.364 | | -5.788 | 3.059 | |  |  |
|  |  |  | 2,000-2,999 | | -1.152 | | -5.631 | 3.326 | |  |  |
|  |  |  | 3,000-3,999 | | -0.448 | | -5.100 | 4.203 | |  |  |
|  |  |  | 4,000-4,999 | | 0.894 | | -4.265 | 6.052 | |  |  |
|  | | | | | | | | | |  |  |
