# Supplementary material for: Our lifestyles are back to ‘normal’, but is our mental health? Longitudinal assessment of psychological distress during the COVID-19 pandemic among Spanish adults: April 2021 to August 2022
Source: PLOS Glob Public Health. 2024 Jul 17;4(7):e0003389. doi: 10.1371/journal.pgph.0003389 (PMC11253917; doi:10.1371/journal.pgph.0003389)
Supplement: S3 Table — (DOCX) [file pgph.0003389.s003.docx]

| **S3 Table.** Bonferroni posthoc comparisons of total stress scores of the sample and demographic characteristics. | | | | | |
| --- | --- | --- | --- | --- | --- |
| **Main Variable** | **Variable level 1** | **Variable level 2** | **Mean Difference** | **95% CI** | |
|  |  |  |  | **Lower Bound** | **Upper Bound** |
| **Age** | 18-34 | 35-60 | 3.424 | **2.164** | **4.685** |
|  |  | 61+ | 6.660 | **5.260** | **8.060** |
|  | 35-60 | 18-34 | -3.424 | **-4.685** | **-2.164** |
|  |  | 61+ | 3.236 | **2.171** | **4.301** |
|  | 61+ | 18-34 | -6.660 | **-8.060** | **-5.260** |
|  |  | 35-60 | -3.236 | **-4.301** | **-2.171** |
| **Civil Status** | Single | Married | 2.308 | **1.114** | **3.503** |
|  |  | Widowed | 2.172 | -0.992 | 5.337 |
|  |  | Divorced or Separated | 2.823 | **0.828** | **4.818** |
|  | Married | Single | -2.308 | **-3.503** | **-1.114** |
|  |  | Widowed | -0.136 | -3.213 | 2.941 |
|  |  | Divorced or Separated | 0.514 | -1.338 | 2.367 |
|  | Widowed | Single | -2.172 | -5.337 | 0.992 |
|  |  | Married | 0.136 | -2.941 | 3.213 |
|  |  | Divorced or Separated | 0.650 | -2.817 | 4.117 |
|  | Divorced or Separated | Single | -2.823 | **-4.818** | **-0.828** |
|  |  | Married | -0.514 | -2.367 | 1.338 |
|  |  | Widowed | -0.650 | -4.117 | 2.817 |
| **Occupation** | Public Sector | Private Sector | -0.597 | -2.409 | 1.215 |
|  |  | Entrepreneur | -1.078 | -4.099 | 1.942 |
|  |  | On Leave | -1.378 | -6.609 | 3.854 |
|  |  | Not Working | -0.664 | -3.065 | 1.736 |
|  |  | Retired | 3.471 | **1.445** | **5.496** |
|  |  | Student | -5.333 | -11.345 | 0.679 |
|  |  | Homemaker | -1.315 | -4.900 | 2.270 |
|  |  | Permanent Incapacity for work | 0.093 | -4.855 | 5.041 |
|  |  | Other economic activity | -1.632 | -8.774 | 5.511 |
|  | Private Sector | Public Sector | 0.597 | -1.215 | 2.409 |
|  |  | Entrepreneur | -0.481 | -3.331 | 2.369 |
|  |  | On Leave | -0.781 | -5.915 | 4.354 |
|  |  | Not Working | -0.067 | -2.249 | 2.115 |
|  |  | Retired | 4.068 | **2.307** | **5.828** |
|  |  | Student | -4.736 | -10.663 | 1.192 |
|  |  | Homemaker | -0.718 | -4.160 | 2.725 |
|  |  | Permanent Incapacity for work | 0.690 | -4.155 | 5.536 |
|  |  | Other economic activity | -1.035 | -8.107 | 6.037 |
|  | Entrepreneur | Public Sector | 1.078 | -1.942 | 4.099 |
|  |  | Private Sector | 0.481 | -2.369 | 3.331 |
|  |  | On Leave | -0.300 | -5.975 | 5.376 |
|  |  | Not Working | 0.414 | -2.842 | 3.670 |
|  |  | Retired | 4.549 | **1.559** | **7.539** |
|  |  | Student | -4.255 | -10.656 | 2.147 |
|  |  | Homemaker | -0.237 | -4.443 | 3.970 |
|  |  | Permanent Incapacity for work | 1.171 | -4.244 | 6.587 |
|  |  | Other economic activity | -0.554 | -8.027 | 6.920 |
|  | On Leave | Public Sector | 1.378 | -3.854 | 6.609 |
|  |  | Private Sector | 0.781 | -4.354 | 5.915 |
|  |  | Entrepreneur | 0.300 | -5.376 | 5.975 |
|  |  | Not Working | 0.714 | -4.657 | 6.084 |
|  |  | Retired | 4.848 | -0.365 | 10.062 |
|  |  | Student | -3.955 | -11.651 | 3.741 |
|  |  | Homemaker | 0.063 | -5.932 | 6.057 |
|  |  | Permanent Incapacity for work | 1.471 | -5.426 | 8.368 |
|  |  | Other economic activity | -0.254 | -8.862 | 8.354 |
|  | Not Working | Public Sector | 0.664 | -1.736 | 3.065 |
|  |  | Private Sector | 0.067 | -2.115 | 2.249 |
|  |  | Entrepreneur | -0.414 | -3.670 | 2.842 |
|  |  | On Leave | -0.714 | -6.084 | 4.657 |
|  |  | Retired | 4.135 | **1.773** | **6.497** |
|  |  | Student | -4.669 | -10.802 | 1.465 |
|  |  | Homemaker | -0.651 | -4.437 | 3.135 |
|  |  | Permanent Incapacity for work | 0.757 | -4.338 | 5.853 |
|  |  | Other economic activity | -0.968 | -8.213 | 6.278 |
|  | Retired | Public Sector | -3.471 | **-5.496** | **-1.445** |
|  |  | Private Sector | -4.068 | **-5.828** | **-2.307** |
|  |  | Entrepreneur | -4.549 | **-7.539** | **-1.559** |
|  |  | On Leave | -4.848 | -10.062 | 0.365 |
|  |  | Not Working | -4.135 | **-6.497** | **-1.773** |
|  |  | Student | -8.804 | **-14.800** | **-2.807** |
|  |  | Homemaker | -4.786 | **-8.345** | **-1.226** |
|  |  | Permanent Incapacity for work | -3.377 | -8.307 | 1.552 |
|  |  | Other economic activity | -5.102 | -12.232 | 2.027 |
|  | Student | Public Sector | 5.333 | -0.679 | 11.345 |
|  |  | Private Sector | 4.736 | -1.192 | 10.663 |
|  |  | Entrepreneur | 4.255 | -2.147 | 10.656 |
|  |  | On Leave | 3.955 | -3.741 | 11.651 |
|  |  | Not Working | 4.669 | -1.465 | 10.802 |
|  |  | Retired | 8.804 | **2.807** | **14.800** |
|  |  | Homemaker | 4.018 | -2.669 | 10.704 |
|  |  | Permanent Incapacity for work | 5.426 | -2.080 | 12.932 |
|  |  | Other economic activity | 3.701 | -5.402 | 12.804 |
|  | Homemaker | Public Sector | 1.315 | -2.270 | 4.900 |
|  |  | Private Sector | 0.718 | -2.725 | 4.160 |
|  |  | Entrepreneur | 0.237 | -3.970 | 4.443 |
|  |  | On Leave | -0.063 | -6.057 | 5.932 |
|  |  | Not Working | 0.651 | -3.135 | 4.437 |
|  |  | Retired | 4.786 | **1.226** | **8.345** |
|  |  | Student | -4.018 | -10.704 | 2.669 |
|  |  | Permanent Incapacity for work | 1.408 | -4.341 | 7.157 |
|  |  | Other economic activity | -0.317 | -8.036 | 7.402 |
|  | Permanent Incapacity for work | Public Sector | -0.093 | -5.041 | 4.855 |
|  |  | Private Sector | -0.690 | -5.536 | 4.155 |
|  |  | Entrepreneur | -1.171 | -6.587 | 4.244 |
|  |  | On Leave | -1.471 | -8.368 | 5.426 |
|  |  | Not Working | -0.757 | -5.853 | 4.338 |
|  |  | Retired | 3.377 | -1.552 | 8.307 |
|  |  | Student | -5.426 | -12.932 | 2.080 |
|  |  | Homemaker | -1.408 | -7.157 | 4.341 |
|  |  | Other economic activity | -1.725 | -10.164 | 6.714 |
|  | Other economic activity | Public Sector | 1.632 | -5.511 | 8.774 |
|  |  | Private Sector | 1.035 | -6.037 | 8.107 |
|  |  | Entrepreneur | 0.554 | -6.920 | 8.027 |
|  |  | On Leave | 0.254 | -8.354 | 8.862 |
|  |  | Not Working | 0.968 | -6.278 | 8.213 |
|  |  | Retired | 5.102 | -2.027 | 12.232 |
|  |  | Student | -3.701 | -12.804 | 5.402 |
|  |  | Homemaker | 0.317 | -7.402 | 8.036 |
|  |  | Permanent Incapacity for work | 1.725 | -6.714 | 10.164 |
| **Income** | Less than 1,000 | 1,000-1,9999 | 2.056 | -0.131 | 4.243 |
|  |  | 2,000-2,999 | 2.268 | -0.027 | 4.564 |
|  |  | 3,000-3,999 | 2.972 | **0.355** | **5.590*** |
|  |  | 4,000-4,999 | 4.314 | **0.876** | **7.752*** |
|  |  | 5,000+ | 3.421 | -1.269 | 8.110 |
|  | 1,000-1,999 | Less than 1,000 | -2.056 | -4.243 | 0.131 |
|  |  | 2,000-2,9999 | 0.212 | -1.476 | 1.899 |
|  |  | 3,000-3,999 | 0.916 | -1.189 | 3.021 |
|  |  | 4,000-4,999 | 2.258 | -0.808 | 5.324 |
|  |  | 5,000+ | 1.364 | -3.059 | 5.788 |
|  | 2,000-2,999 | Less than 1,000 | -2.268 | -4.564 | 0.027 |
|  |  | 1,000-1,9999 | -0.212 | -1.899 | 1.476 |
|  |  | 3,000-3,999 | 0.704 | -1.513 | 2.922 |
|  |  | 4,000-4,999 | 2.046 | -1.098 | 5.190 |
|  |  | 5,000+ | 1.152 | -3.326 | 5.631 |
|  | 3,000-3,999 | Less than 1,000 | -2.972 | **-5.590** | **-0.355*** |
|  |  | 1,000-1,9999 | -0.916 | -3.021 | 1.189 |
|  |  | 2,000-2,999 | -0.704 | -2.922 | 1.513 |
|  |  | 4,000-4,999 | 1.342 | -2.045 | 4.728 |
|  |  | 5,000+ | 0.448 | -4.203 | 5.100 |
|  | 4,000-4,999 | Less than 1,000 | -4.314 | **-7.752** | **-0.876*** |
|  |  | 1,000-1,9999 | -2.258 | -5.324 | 0.808 |
|  |  | 2,000-2,999 | -2.046 | -5.190 | 1.098 |
|  |  | 3,000-3,999 | -1.342 | -4.728 | 2.045 |
|  |  | 5,000+ | -0.894 | -6.052 | 4.265 |
|  | 5,000+ | Less than 1,000 | -3.421 | -8.110 | 1.269 |
|  |  | 1,000-1,9999 | -1.364 | -5.788 | 3.059 |
|  |  | 2,000-2,999 | -1.152 | -5.631 | 3.326 |
|  |  | 3,000-3,999 | -0.448 | -5.100 | 4.203 |
|  |  | 4,000-4,999 | 0.894 | -4.265 | 6.052 |
|  | | | | | |
